# Bis[triaqua­(1*H*-1,2,4-triazole-3,5-dicarboxyl­ato-κ^2^ *O* ^3^,*N* ^4^)copper(II)] di-μ-aqua-bis­[diaqua­(1*H*-1,2,4-triazole-3,5-dicarboxyl­ato-κ^2^ *O* ^3^,*N* ^4^)copper(II)]

**DOI:** 10.1107/S1600536809026993

**Published:** 2009-07-15

**Authors:** Li-Xia Xie, Xin Li, Pu-Hui Xie, Qiu Jin

**Affiliations:** a College of Sciences, Henan Agricultural University, Zhengzhou, Henan 450002, People’s Republic of China

## Abstract

In the title compound, [Cu(C_4_HN_3_O_4_)(H_2_O)_3_]_2_[Cu_2_(C_4_HN_3_O_4_)_2_(H_2_O)_6_], both monomeric and dimeric mol­ecules are present in the solid state. In the monomeric compound, the Cu^II^ atom is five-coordinated in a square-pyramidal configuration by one O atom and one N atom from one 1*H*-1,2,4-triazole-3,5-dicarboxyl­ate (TZDCA^2−^) ligand and three O atoms from water mol­ecules. In the centrosymmetric binuclear complex, each Cu^II^ atom is six-coordinated in an octa­hedral geometry by one O atom and one N atom from one TZDCA^2−^ ligand and four O atoms from water mol­ecules, two of which bridge the Cu^II^ atoms. In the structure, there are intra­molecular O—H⋯O and N—H⋯O hydrogen bonds, and in the crystal, inter­molecular O—H⋯O, O—H⋯N and N—H⋯O hydrogen bonds link symmetry-related mol­ecules, forming a three-dimensional supra­molecular structure.

## Related literature

For related structures, see: Billing *et al.* (1970 ▶); Ouellette *et al.* (2006*a* ▶,*b* ▶, 2007 ▶); Zhai *et al.* (2007 ▶). For the preparation of 1,2,4,-triazole-3,5-dicarboxylic acid, see: Baitalik *et al.* (2004 ▶).
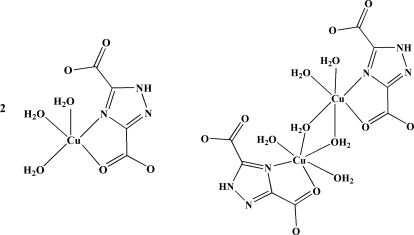


## Experimental

### 

#### Crystal data

[Cu(C_4_HN_3_O_4_)(H_2_O)_3_]_2_[Cu_2_(C_4_HN_3_O_4_)_2_(H_2_O)_6_]
*M* *_r_* = 1090.70
Monoclinic, 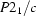

*a* = 12.056 (2) Å
*b* = 11.432 (2) Å
*c* = 14.958 (3) Å
β = 123.65 (3)°
*V* = 1716.1 (5) Å^3^
*Z* = 2
Mo *K*α radiation
μ = 2.57 mm^−1^
*T* = 293 K
0.10 × 0.10 × 0.08 mm

#### Data collection

Mercury CCD diffractometer
Absorption correction: multi-scan (*CrystalClear*; Rigaku, 2000 ▶) *T*_min_ = 0.783, *T*_max_ = 0.821
16043 measured reflections
3020 independent reflections
2866 reflections with *I* > 2σ(*I*)
*R*_int_ = 0.051

#### Refinement

*R*[*F* ^2^ > 2σ(*F* ^2^)] = 0.046
*wR*(*F* ^2^) = 0.115
*S* = 1.17
3020 reflections
272 parameters
H-atom parameters constrained
Δρ_max_ = 0.64 e Å^−3^
Δρ_min_ = −0.54 e Å^−3^

### 

Data collection: *CrystalClear* (Rigaku, 2000 ▶); cell refinement: *CrystalClear*; data reduction: *CrystalClear*; program(s) used to solve structure: *SHELXS97* (Sheldrick, 2008 ▶); program(s) used to refine structure: *SHELXL97* (Sheldrick, 2008 ▶); molecular graphics: *SHELXTL* (Sheldrick, 2008 ▶); software used to prepare material for publication: *SHELXTL*.

## Supplementary Material

Crystal structure: contains datablocks global, I. DOI: 10.1107/S1600536809026993/su2127sup1.cif

Structure factors: contains datablocks I. DOI: 10.1107/S1600536809026993/su2127Isup2.hkl

Additional supplementary materials:  crystallographic information; 3D view; checkCIF report

## Figures and Tables

**Table 1:** Hydrogen-bond geometry (Å, °)

*D*—H⋯*A*	*D*—H	H⋯*A*	*D*⋯*A*	*D*—H⋯*A*
N2—H2⋯O6^i^	0.85	1.87	2.709 (6)	169
N5—H5⋯O8	0.85	2.46	2.773 (5)	103
N5—H5⋯O2^ii^	0.85	1.89	2.655 (7)	148
O9—H9*A*⋯O1^iii^	0.85	2.50	3.221 (5)	144
O9—H9*A*⋯O2^iii^	0.85	2.49	3.256 (5)	150
O9—H9*B*⋯O7^ii^	0.85	1.85	2.596 (5)	145
O10—H10*A*⋯O2^ii^	0.85	2.02	2.798 (6)	151
O10—H10*B*⋯O5^iv^	0.85	2.59	3.103 (6)	120
O10—H10*B*⋯N1^ii^	0.85	2.10	2.863 (5)	149
O11—H11*A*⋯O8^ii^	0.85	1.84	2.650 (5)	160
O11—H11*B*⋯O3	0.85	1.83	2.662 (6)	167
O12—H12*A*⋯O6^i^	0.83	2.15	2.811 (6)	137
O12—H12*B*⋯O1^v^	0.83	2.49	3.111 (6)	132
O12—H12*B*⋯N4^i^	0.83	2.19	2.821 (5)	133
O13—H13*A*⋯O4^i^	0.85	1.89	2.714 (5)	164
O13—H13*B*⋯O7	0.85	1.81	2.654 (7)	172
O14—H14*A*⋯O10^v^	0.85	1.86	2.686 (5)	163
O14—H14*B*⋯O3^i^	0.85	1.76	2.599 (5)	169

Symmetry codes: (i) 
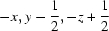
; (ii) 
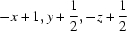
; (iii) 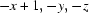; (iv) 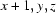; (v) 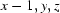.

**Fig. 1.**
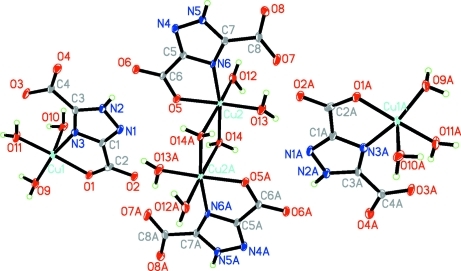
A view of the molecular structure of the title compound, showing 30% probability displacement ellipsoids and the atom-numbering (Symmetry code: (A) = -x, -y, -z+1).

**Fig. 2.**
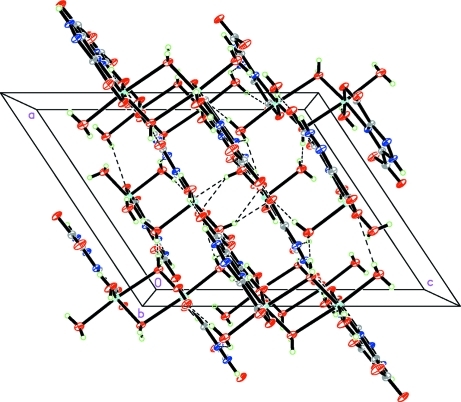
A view along the b axis of the crystal packing of the title compound, showing the hydrogen bonds as pale-blue dashed lines (see Table 1 for details).

## supplementary crystallographic information

### Comment

As a ligand with multiple coordination sites, 1,2,4-triazole has been shown to be good organic linker in the generation of structurally versatile metal-organic frameworks. It can bridge different metal centers to afford coordination polymers that exhibit extraordinary structural diversity and facile accessibility of functionalized new magnetic materials (Ouellette *et al.*, 2006a; Ouellette *et al.*, 2006b; Ouellette *et al.*, 2007; Zhai *et al.*, 2007). Furthermore, functional groups such as carboxylate, amino and pyridyl, can be introduced in 1,2,4-triazole, which makes its chemistry more abundant and complex. Encouraged by this aspect we selected a simple bifunctional ligand containing both 1,2,4-triazole and carboxylate groups, 1,2,4,-triazole-3,5-dicarboxylic acid, to study its coordination chemistry. As a result, we report herein on the crystal structure of the title compound.

The asymmetric unit of the title compound (Fig. 1) contains a monomeric complex (Scheme 1) and half of a centrosymmetric binuclear complex (Scheme 2). In the mononuclear complex atom Cu1 is five-coordinated, by one N atom and one O atom from one TZDCA^2-^ ligand and three water molecules, and has a slightly distorted square pyramidal geometry. In the binuclear dimeric complex the Cu2 ions adopt an octahedral coordination geometry, where one N-atom and one O-atom from one TZDCA^2-^ ligand and two water molecules are in the equatorial plane, while the apical positions are occupied by water molecules. Water O14 acts as a bridge to form a four-membered Cu2/O14/O14A/Cu2A ring (Symmetry code: (A)= -x, -y, -z+1). The bond length Cu2–O14A = 2.617 (3) Å, indicates a weak coordination interaction (Billing *et al.*, 1970). Each TZDCA^2-^ is deprotonated and acts as a bidentate ligand.

In the crystal structure there intra- and inter-molecular hydrogen bonds (Table 1), which consolidate the structure (Fig. 2).

### Experimental

All solvents and chemicals were of analytical grade and were used without further purification. Ligand 1,2,4,-triazole-3,5-dicarboxylic acid was prepared by the literature method (Baitalik *et al.*, 2004). The title compound was synthesized as follows: 1,2,4,-triazole-3,5-dicarboxylic acid (0.5 mmol) was added to 5 cm^3^ water and the resulting solution was adjusted to a pH of 7.0, using an aqueous solution of triethylamine. CuSO_4_(0.5 mmol) was then added to the above solution, and the mixture was stirred for 30 min and then filtered. The filtrate was left to evaporate slowly in air. After six days, blue single crystals suitable for X-ray analysis were obtained. Anal. Calcd (%) for C_4_H_7_CuN_3_O_7_: C, 17.62; H, 2.59; N, 15.41. Found (%): C, 17.73; H, 2.45; N, 15.52.

### Refinement

The H atoms were included in calculated positions and treated as riding atoms: O–H = 0.83 - 0.85 Å and N–H = 0.85 Å, with U_iso_(H) = 1.5U_eq_(parent O-atom) and 1.2U_eq_(parent N-atom).

### Crystal data

**Table:**

[Cu(C_4_HN_3_O_4_)(H_2_O)_3_]_2_[Cu_2_(C_4_HN_3_O_4_)_2_(H_2_O)_6_]	*F*(000) = 1096
*M**_r_* = 1090.70	*D*_x_ = 2.111 Mg m^−^^3^
Monoclinic, *P*2_1_/*c*	Mo *K*α radiation, λ = 0.71073 Å
Hall symbol: -P 2ybc	Cell parameters from 3290 reflections
*a* = 12.056 (2) Å	θ = 2.0–31.0°
*b* = 11.432 (2) Å	µ = 2.57 mm^−^^1^
*c* = 14.958 (3) Å	*T* = 293 K
β = 123.65 (3)°	Prism, blue
*V* = 1716.1 (5) Å^3^	0.10 × 0.10 × 0.08 mm
*Z* = 2	

### Data collection

**Table:**

Mercury CCD diffractometer	3020 independent reflections
Radiation source: fine-focus sealed tube	2866 reflections with *I* > 2σ(*I*)
graphite	*R*_int_ = 0.051
ω scans	θ_max_ = 25.0°, θ_min_ = 2.0°
Absorption correction: multi-scan (*CrystalClear*; Rigaku, 2000)	*h* = −14→14
*T*_min_ = 0.783, *T*_max_ = 0.821	*k* = −13→13
16043 measured reflections	*l* = −17→17

### Refinement

**Table:**

Refinement on *F*^2^	Primary atom site location: structure-invariant direct methods
Least-squares matrix: full	Secondary atom site location: difference Fourier map
*R*[*F*^2^ > 2σ(*F*^2^)] = 0.046	Hydrogen site location: inferred from neighbouring sites
*wR*(*F*^2^) = 0.115	H-atom parameters constrained
*S* = 1.17	*w* = 1/[σ^2^(*F*_o_^2^) + (0.0397*P*)^2^ + 6.4925*P*] where *P* = (*F*_o_^2^ + 2*F*_c_^2^)/3
3020 reflections	(Δ/σ)_max_ < 0.001
272 parameters	Δρ_max_ = 0.64 e Å^−^^3^
0 restraints	Δρ_min_ = −0.54 e Å^−^^3^

### Special details

**Table:**

Geometry. Bond distances, angles etc. have been calculated using the rounded fractional coordinates. All su's are estimated from the variances of the (full) variance-covariance matrix. The cell esds are taken into account in the estimation of distances, angles and torsion angles
Refinement. Refinement of *F*^2^ against ALL reflections. The weighted *R*-factor *wR* and goodness of fit *S* are based on *F*^2^, conventional *R*-factors *R* are based on *F*, with *F* set to zero for negative *F*^2^. The threshold expression of *F*^2^ > σ(*F*^2^) is used only for calculating *R*-factors(gt) *etc*. and is not relevant to the choice of reflections for refinement. *R*-factors based on *F*^2^ are statistically about twice as large as those based on *F*, and *R*- factors based on ALL data will be even larger.

### Fractional atomic coordinates and isotropic or equivalent isotropic displacement parameters (Å^2^)

**Table:**

	*x*	*y*	*z*	*U*_iso_*/*U*_eq_	
Cu2	−0.00361 (5)	0.00777 (4)	0.38176 (4)	0.0204 (2)	
O5	−0.0608 (3)	0.1733 (3)	0.3706 (3)	0.0251 (10)	
O6	0.0026 (3)	0.3562 (3)	0.3659 (3)	0.0273 (10)	
O7	0.2747 (4)	−0.1431 (3)	0.3948 (3)	0.0386 (13)	
O8	0.4173 (3)	−0.0451 (3)	0.3711 (3)	0.0322 (11)	
O12	−0.1496 (3)	−0.0099 (3)	0.1914 (3)	0.0299 (10)	
O13	0.0607 (3)	−0.1507 (3)	0.4033 (3)	0.0401 (13)	
O14	−0.1385 (3)	−0.0368 (3)	0.4098 (2)	0.0229 (9)	
N4	0.2191 (4)	0.2525 (3)	0.3602 (3)	0.0239 (11)	
N5	0.2979 (4)	0.1643 (3)	0.3662 (3)	0.0224 (11)	
N6	0.1440 (3)	0.0784 (3)	0.3743 (3)	0.0179 (10)	
C5	0.1271 (4)	0.1964 (4)	0.3656 (3)	0.0194 (12)	
C6	0.0143 (4)	0.2496 (4)	0.3669 (3)	0.0195 (12)	
C7	0.2535 (4)	0.0614 (4)	0.3741 (3)	0.0190 (12)	
C8	0.3216 (4)	−0.0531 (4)	0.3805 (4)	0.0234 (12)	
Cu1	0.51531 (5)	0.10043 (5)	0.13087 (5)	0.0233 (2)	
O1	0.5666 (3)	−0.0657 (3)	0.1367 (3)	0.0280 (10)	
O2	0.4995 (3)	−0.2483 (3)	0.1359 (3)	0.0271 (10)	
O3	0.2329 (3)	0.2532 (3)	0.1120 (3)	0.0302 (10)	
O4	0.0728 (3)	0.1515 (3)	0.1110 (3)	0.0310 (10)	
O9	0.6516 (3)	0.1440 (3)	0.1069 (3)	0.0302 (10)	
O10	0.6585 (3)	0.1163 (3)	0.3205 (3)	0.0316 (10)	
O11	0.4531 (3)	0.2599 (3)	0.1113 (3)	0.0351 (10)	
N1	0.2712 (4)	−0.1437 (3)	0.1204 (3)	0.0253 (11)	
N2	0.1924 (3)	−0.0561 (3)	0.1154 (3)	0.0229 (11)	
N3	0.3567 (3)	0.0310 (3)	0.1222 (3)	0.0210 (11)	
C1	0.3685 (4)	−0.0882 (4)	0.1249 (4)	0.0217 (12)	
C2	0.4882 (4)	−0.1371 (4)	0.1318 (3)	0.0215 (11)	
C3	0.2427 (4)	0.0478 (4)	0.1159 (3)	0.0199 (12)	
C4	0.1757 (4)	0.1610 (4)	0.1129 (4)	0.0212 (12)	
H5	0.36710	0.16420	0.36410	0.0270*	
H12A	−0.09350	−0.01230	0.17580	0.0450*	
H12B	−0.19850	−0.06340	0.18780	0.0450*	
H13A	0.01660	−0.21390	0.38700	0.0600*	
H13B	0.12470	−0.14650	0.39470	0.0600*	
H14A	−0.21270	−0.00110	0.37720	0.0340*	
H14B	−0.16800	−0.10650	0.39590	0.0340*	
H2	0.12460	−0.08260	0.11240	0.0280*	
H9A	0.61880	0.14370	0.03990	0.0460*	
H9B	0.68010	0.21210	0.13200	0.0460*	
H10A	0.60750	0.13340	0.34120	0.0480*	
H10B	0.70320	0.17910	0.33550	0.0480*	
H11A	0.50060	0.32140	0.13270	0.0530*	
H11B	0.38230	0.26930	0.11000	0.0530*	

### Atomic displacement parameters (Å^2^)

**Table:**

	*U*^11^	*U*^22^	*U*^33^	*U*^12^	*U*^13^	*U*^23^
Cu2	0.0215 (3)	0.0096 (3)	0.0376 (3)	0.0003 (2)	0.0211 (3)	0.0019 (2)
O5	0.0252 (16)	0.0143 (15)	0.047 (2)	−0.0007 (12)	0.0270 (15)	0.0022 (13)
O6	0.0313 (17)	0.0137 (16)	0.049 (2)	0.0024 (13)	0.0298 (17)	0.0010 (14)
O7	0.040 (2)	0.0133 (16)	0.082 (3)	0.0048 (15)	0.046 (2)	0.0052 (17)
O8	0.0322 (18)	0.0224 (17)	0.059 (2)	0.0065 (14)	0.0359 (18)	0.0019 (16)
O12	0.0283 (17)	0.0197 (16)	0.046 (2)	−0.0011 (13)	0.0232 (16)	−0.0058 (14)
O13	0.038 (2)	0.0178 (17)	0.084 (3)	0.0009 (15)	0.046 (2)	0.0049 (17)
O14	0.0207 (15)	0.0112 (14)	0.0397 (18)	−0.0004 (12)	0.0185 (14)	0.0021 (13)
N4	0.0247 (19)	0.0134 (18)	0.042 (2)	0.0041 (15)	0.0237 (18)	0.0021 (16)
N5	0.0193 (17)	0.0155 (18)	0.041 (2)	0.0007 (14)	0.0221 (17)	−0.0007 (16)
N6	0.0217 (18)	0.0070 (16)	0.0291 (19)	0.0020 (13)	0.0166 (16)	0.0015 (14)
C5	0.021 (2)	0.013 (2)	0.030 (2)	−0.0005 (16)	0.0177 (19)	−0.0002 (17)
C6	0.022 (2)	0.012 (2)	0.028 (2)	−0.0005 (17)	0.0161 (19)	−0.0009 (17)
C7	0.021 (2)	0.013 (2)	0.025 (2)	0.0027 (17)	0.0140 (18)	0.0022 (16)
C8	0.026 (2)	0.015 (2)	0.031 (2)	0.0035 (18)	0.017 (2)	−0.0009 (18)
Cu1	0.0210 (3)	0.0137 (3)	0.0399 (4)	−0.0015 (2)	0.0198 (3)	0.0011 (2)
O1	0.0277 (17)	0.0152 (16)	0.052 (2)	0.0029 (13)	0.0290 (16)	−0.0010 (14)
O2	0.0264 (16)	0.0140 (16)	0.052 (2)	0.0028 (13)	0.0287 (16)	0.0021 (14)
O3	0.0283 (17)	0.0115 (15)	0.056 (2)	0.0022 (13)	0.0267 (17)	0.0002 (14)
O4	0.0278 (17)	0.0203 (17)	0.052 (2)	0.0039 (13)	0.0266 (17)	0.0024 (15)
O9	0.0302 (17)	0.0166 (16)	0.053 (2)	−0.0025 (13)	0.0289 (17)	0.0021 (15)
O10	0.0275 (17)	0.0252 (17)	0.045 (2)	0.0013 (14)	0.0219 (16)	−0.0071 (15)
O11	0.0296 (17)	0.0144 (16)	0.069 (2)	−0.0006 (13)	0.0321 (18)	0.0027 (16)
N1	0.0240 (19)	0.0114 (18)	0.047 (2)	−0.0011 (14)	0.0237 (19)	−0.0015 (16)
N2	0.0178 (18)	0.0157 (19)	0.039 (2)	0.0003 (14)	0.0182 (17)	0.0012 (16)
N3	0.0189 (17)	0.0170 (18)	0.032 (2)	0.0016 (14)	0.0172 (16)	0.0017 (15)
C1	0.023 (2)	0.013 (2)	0.032 (2)	0.0018 (17)	0.017 (2)	0.0003 (17)
C2	0.0107 (19)	0.026 (2)	0.024 (2)	0.0070 (17)	0.0072 (18)	−0.0032 (18)
C3	0.022 (2)	0.013 (2)	0.026 (2)	−0.0004 (17)	0.0142 (19)	0.0021 (17)
C4	0.017 (2)	0.018 (2)	0.030 (2)	0.0040 (17)	0.0139 (19)	0.0020 (18)

### Geometric parameters (Å, °)

**Table:**

Cu2—O5	1.989 (4)	O4—C4	1.230 (7)
Cu2—O12	2.386 (4)	O9—H9A	0.8500
Cu2—O13	1.926 (4)	O9—H9B	0.8500
Cu2—O14	1.959 (4)	O10—H10B	0.8500
Cu2—N6	2.013 (4)	O10—H10A	0.8500
Cu2—O14^i^	2.617 (3)	O11—H11A	0.8500
Cu1—N3	2.007 (4)	O11—H11B	0.8500
Cu1—O9	1.931 (4)	N4—N5	1.354 (6)
Cu1—O10	2.373 (4)	N4—C5	1.322 (7)
Cu1—O1	1.984 (4)	N5—C7	1.325 (6)
Cu1—O11	1.931 (4)	N6—C7	1.336 (7)
O5—C6	1.280 (6)	N6—C5	1.360 (6)
O6—C6	1.226 (6)	N5—H5	0.8500
O7—C8	1.248 (6)	N1—N2	1.354 (6)
O8—C8	1.241 (7)	N1—C1	1.302 (8)
O12—H12B	0.8300	N2—C3	1.332 (6)
O12—H12A	0.8300	N3—C1	1.368 (6)
O13—H13A	0.8500	N3—C3	1.338 (7)
O13—H13B	0.8500	N2—H2	0.8500
O14—H14A	0.8500	C5—C6	1.500 (7)
O14—H14B	0.8500	C7—C8	1.520 (7)
O1—C2	1.220 (6)	C1—C2	1.497 (8)
O2—C2	1.276 (6)	C3—C4	1.513 (7)
O3—C4	1.264 (6)		
			
O5—Cu2—O12	89.33 (14)	Cu1—O10—H10B	108.00
O5—Cu2—O13	175.94 (16)	Cu1—O10—H10A	105.00
O5—Cu2—O14	88.58 (17)	Cu1—O11—H11B	115.00
O5—Cu2—N6	83.67 (16)	H11A—O11—H11B	111.00
O5—Cu2—O14^i^	86.96 (14)	Cu1—O11—H11A	127.00
O12—Cu2—O13	94.73 (14)	N5—N4—C5	102.5 (4)
O12—Cu2—O14	94.74 (13)	N4—N5—C7	111.3 (5)
O12—Cu2—N6	93.33 (15)	Cu2—N6—C7	147.9 (3)
O12—Cu2—O14^i^	174.72 (14)	Cu2—N6—C5	108.3 (3)
O13—Cu2—O14	91.41 (17)	C5—N6—C7	103.9 (4)
O13—Cu2—N6	95.76 (17)	N4—N5—H5	132.00
O13—Cu2—O14^i^	89.02 (14)	C7—N5—H5	117.00
O14—Cu2—N6	168.74 (14)	N2—N1—C1	103.1 (4)
O14—Cu2—O14^i^	81.43 (12)	N1—N2—C3	110.9 (4)
O14^i^—Cu2—N6	90.00 (14)	Cu1—N3—C1	108.2 (3)
O9—Cu1—N3	165.50 (16)	Cu1—N3—C3	148.5 (3)
O1—Cu1—O9	88.77 (17)	C1—N3—C3	103.4 (4)
O1—Cu1—O10	91.04 (14)	C3—N2—H2	138.00
O1—Cu1—O11	174.72 (16)	N1—N2—H2	111.00
O1—Cu1—N3	83.50 (17)	N4—C5—N6	113.6 (5)
O9—Cu1—O10	94.13 (16)	N6—C5—C6	119.3 (4)
O9—Cu1—O11	91.57 (17)	N4—C5—C6	127.0 (4)
O11—Cu1—N3	95.02 (17)	O5—C6—O6	126.8 (5)
O10—Cu1—O11	94.20 (14)	O5—C6—C5	113.1 (4)
O10—Cu1—N3	98.25 (15)	O6—C6—C5	120.1 (5)
Cu2—O5—C6	115.6 (4)	N5—C7—N6	108.6 (4)
Cu2—O14—Cu2^i^	98.57 (13)	N6—C7—C8	128.7 (4)
Cu2—O12—H12A	99.00	N5—C7—C8	122.7 (5)
Cu2—O12—H12B	100.00	O8—C8—C7	115.7 (4)
H12A—O12—H12B	128.00	O7—C8—O8	128.2 (5)
Cu2—O13—H13A	129.00	O7—C8—C7	116.1 (5)
Cu2—O13—H13B	104.00	N1—C1—N3	114.1 (5)
H13A—O13—H13B	119.00	N1—C1—C2	128.9 (4)
Cu2—O14—H14B	118.00	N3—C1—C2	117.0 (5)
H14A—O14—H14B	98.00	O1—C2—O2	127.2 (5)
Cu2^i^—O14—H14A	114.00	O1—C2—C1	116.1 (4)
Cu2—O14—H14A	119.00	O2—C2—C1	116.6 (5)
Cu2^i^—O14—H14B	109.00	N3—C3—C4	129.5 (4)
Cu1—O1—C2	115.2 (4)	N2—C3—N3	108.6 (4)
Cu1—O9—H9B	109.00	N2—C3—C4	121.9 (5)
H9A—O9—H9B	110.00	O3—C4—C3	115.4 (5)
Cu1—O9—H9A	109.00	O4—C4—C3	116.1 (4)
H10A—O10—H10B	101.00	O3—C4—O4	128.5 (5)
			
O12—Cu2—O5—C6	−95.3 (3)	C5—N6—C7—C8	−179.2 (4)
O14—Cu2—O5—C6	170.0 (3)	Cu2—N6—C5—N4	179.9 (3)
N6—Cu2—O5—C6	−1.9 (3)	C7—N6—C5—N4	−0.1 (5)
O14^i^—Cu2—O5—C6	88.5 (3)	C5—N6—C7—N5	0.3 (4)
O5—Cu2—O14—Cu2^i^	−87.14 (15)	Cu2—N6—C5—C6	2.0 (4)
O12—Cu2—O14—Cu2^i^	−176.35 (12)	Cu2—N6—C7—C8	0.9 (8)
O13—Cu2—O14—Cu2^i^	88.80 (15)	Cu2—N6—C7—N5	−179.7 (4)
O14^i^—Cu2—O14—Cu2^i^	−0.02 (13)	C7—N6—C5—C6	−178.0 (3)
O5—Cu2—N6—C5	−0.2 (3)	C1—N1—N2—C3	0.6 (5)
O5—Cu2—N6—C7	179.7 (6)	N2—N1—C1—N3	−0.5 (5)
O12—Cu2—N6—C5	88.8 (3)	N2—N1—C1—C2	179.9 (5)
O12—Cu2—N6—C7	−91.3 (6)	N1—N2—C3—N3	−0.5 (5)
O13—Cu2—N6—C5	−176.2 (3)	N1—N2—C3—C4	−178.9 (4)
O13—Cu2—N6—C7	3.8 (6)	C3—N3—C1—C2	179.9 (4)
O14^i^—Cu2—N6—C5	−87.1 (3)	C3—N3—C1—N1	0.2 (5)
O14^i^—Cu2—N6—C7	92.8 (6)	Cu1—N3—C1—N1	179.6 (3)
O5—Cu2—O14^i^—Cu2^i^	89.02 (17)	C1—N3—C3—C4	178.4 (4)
O13—Cu2—O14^i^—Cu2^i^	−91.57 (18)	Cu1—N3—C3—C4	−0.5 (9)
O14—Cu2—O14^i^—Cu2^i^	0.02 (16)	Cu1—N3—C1—C2	−0.7 (5)
N6—Cu2—O14^i^—Cu2^i^	172.68 (16)	Cu1—N3—C3—N2	−178.7 (4)
O11—Cu1—N3—C3	−6.2 (6)	C1—N3—C3—N2	0.2 (4)
O9—Cu1—O1—C2	−166.9 (3)	N6—C5—C6—O6	175.4 (4)
O10—Cu1—O1—C2	99.0 (3)	N4—C5—C6—O6	−2.2 (6)
N3—Cu1—O1—C2	0.8 (3)	N6—C5—C6—O5	−3.6 (5)
O1—Cu1—N3—C1	0.0 (3)	N4—C5—C6—O5	178.8 (4)
O1—Cu1—N3—C3	178.9 (6)	N6—C7—C8—O7	−5.1 (7)
O10—Cu1—N3—C1	−90.1 (3)	N6—C7—C8—O8	175.1 (4)
O10—Cu1—N3—C3	88.8 (6)	N5—C7—C8—O8	−4.3 (6)
O11—Cu1—N3—C1	174.9 (3)	N5—C7—C8—O7	175.5 (4)
Cu2—O5—C6—O6	−175.7 (4)	N1—C1—C2—O1	−178.9 (5)
Cu2—O5—C6—C5	3.3 (4)	N3—C1—C2—O1	1.5 (6)
Cu1—O1—C2—C1	−1.4 (5)	N3—C1—C2—O2	179.0 (4)
Cu1—O1—C2—O2	−178.6 (3)	N1—C1—C2—O2	−1.4 (7)
C5—N4—N5—C7	0.4 (4)	N2—C3—C4—O3	−178.9 (4)
N5—N4—C5—C6	177.5 (4)	N2—C3—C4—O4	0.7 (6)
N5—N4—C5—N6	−0.2 (5)	N3—C3—C4—O3	3.1 (7)
N4—N5—C7—N6	−0.4 (5)	N3—C3—C4—O4	−177.4 (4)
N4—N5—C7—C8	179.1 (4)		

Symmetry codes: (i) −*x*, −*y*, −*z*+1.

### Hydrogen-bond geometry (Å, °)

**Table:**

*D*—H···*A*	*D*—H	H···*A*	*D*···*A*	*D*—H···*A*
N2—H2···O6^ii^	0.85	1.87	2.709 (6)	169
N5—H5···O8	0.85	2.46	2.773 (5)	103
N5—H5···O2^iii^	0.85	1.89	2.655 (7)	148
O9—H9A···O1^iv^	0.85	2.50	3.221 (5)	144
O9—H9A···O2^iv^	0.85	2.49	3.256 (5)	150
O9—H9B···O7^iii^	0.85	1.85	2.596 (5)	145
O10—H10A···O2^iii^	0.85	2.02	2.798 (6)	151
O10—H10B···O5^v^	0.85	2.59	3.103 (6)	120
O10—H10B···N1^iii^	0.85	2.10	2.863 (5)	149
O11—H11A···O8^iii^	0.85	1.84	2.650 (5)	160
O11—H11B···O3	0.85	1.83	2.662 (6)	167
O12—H12A···O6^ii^	0.83	2.15	2.811 (6)	137
O12—H12B···O1^vi^	0.83	2.49	3.111 (6)	132
O12—H12B···N4^ii^	0.83	2.19	2.821 (5)	133
O13—H13A···O4^ii^	0.85	1.89	2.714 (5)	164
O13—H13B···O7	0.85	1.81	2.654 (7)	172
O14—H14A···O10^vi^	0.85	1.86	2.686 (5)	163
O14—H14B···O3^ii^	0.85	1.76	2.599 (5)	169

Symmetry codes: (ii) −*x*, *y*−1/2, −*z*+1/2; (iii) −*x*+1, *y*+1/2, −*z*+1/2; (iv) −*x*+1, −*y*, −*z*; (v) *x*+1, *y*, *z*; (vi) *x*−1, *y*, *z*.

## References

[bb1] Baitalik, S., Dutta, B. & Nag, K. (2004). *Polyhedron*, **23**, 913–919.

[bb2] Billing, D. E., Hathaway, B. J. & Nivholls, P. J. (1970). *J. Chem. Soc. A*, pp. 1877–1881.

[bb3] Ouellette, W., Galan Mascaros, J. R., Dunbar, K. R. & Zubieta, J. (2006*a*). *Inorg. Chem.* **45**, 1909–1911. 10.1021/ic051992i 16499350

[bb4] Ouellette, W., Hudson, B. & Zubieta, J. (2007). *Inorg. Chem.* **46**, 4887–4904. 10.1021/ic062269a 17497849

[bb5] Ouellette, W., Prosvirin, A. V., Chieffo, V., Dunbar, K. R., Hudson, B. & Zubieta, J. (2006*b*). *Inorg. Chem.* **45**, 9346–9366. 10.1021/ic061102e 17083234

[bb6] Rigaku (2000). *CrystalClear.* Rigaku Corporation, Tokyo, Japan.

[bb7] Sheldrick, G. M. (2008). *Acta Cryst.* A**64**, 112–122. 10.1107/S0108767307043930 18156677

[bb8] Zhai, Q. G., Lu, C. Z., Wu, X. Y. & Batten, S. R. (2007). *Cryst. Growth Des.* **7**, 2332–2342.

